# Noble Metal Composite Porous Silk Fibroin Aerogel Fibers

**DOI:** 10.3390/ma12060894

**Published:** 2019-03-18

**Authors:** Alexander N. Mitropoulos, F. John Burpo, Chi K. Nguyen, Enoch A. Nagelli, Madeline Y. Ryu, Jenny Wang, R. Kenneth Sims, Kamil Woronowicz, J. Kenneth Wickiser

**Affiliations:** 1 Department of Chemistry and Life Science, United States Military Academy, West Point, NY 10996, USA; chi.nguyen@westpoint.edu (C.K.N.); enoch.nagelli@westpoint.edu (E.A.N.); madeline.ryu@westpoint.edu (M.Y.R.); jenny.wang@westpoint.edu (J.W.); kamil.woronowicz@westpoint.edu (K.W.); ken.wickiser@westpoint.edu (J.K.W.); 2 Department of Mathematical Sciences, United States Military Academy, West Point, NY 10996, USA; 3 Department of Civil and Mechanical Engineering, United States Military Academy, West Point, NY 10996, USA; robert.sims@westpoint.edu

**Keywords:** biopolymer, silk fibroin, aerogel, fiber, nanomaterials, nanoparticles, noble metals, gold, platinum, palladium

## Abstract

Nobel metal composite aerogel fibers made from flexible and porous biopolymers offer a wide range of applications, such as in catalysis and sensing, by functionalizing the nanostructure. However, producing these composite aerogels in a defined shape is challenging for many protein-based biopolymers, especially ones that are not fibrous proteins. Here, we present the synthesis of silk fibroin composite aerogel fibers up to 2 cm in length and a diameter of ~300 μm decorated with noble metal nanoparticles. Lyophilized silk fibroin dissolved in hexafluoro-2-propanol (HFIP) was cast in silicon tubes and physically crosslinked with ethanol to produce porous silk gels. Composite silk aerogel fibers with noble metals were created by equilibrating the gels in noble metal salt solutions reduced with sodium borohydride, followed by supercritical drying. These porous aerogel fibers provide a platform for incorporating noble metals into silk fibroin materials, while also providing a new method to produce porous silk fibers. Noble metal silk aerogel fibers can be used for biological sensing and energy storage applications.

## 1. Introduction

Biopolymers provide unique applications in advanced technology where degradation combined with natural materials are required. In nature, biopolymers take several forms, such as films, fibers, gels, and sponges, which are optimized for their required applications [1,2]. However, producing the equivalent forms with the desired qualities in regenerated biopolymers has been challenging, especially making fibers with controlled diameters and porosity [2]. Nevertheless, working with regenerated biopolymer solutions can enhance properties such as the tensile strength and porosity [3]. Furthermore, regenerated biopolymer solutions can be used as a structural network that can be combined with other materials to synthesize composites not found in nature [4,5]. Starting with biopolymers dissolved in polar solvents, such as water or alcohols, can be useful in providing varied biopolymer conformational folding to enhance the desired properties. 

Silk fibroin, purified from the *Bombyx mori* silk caterpillar, is a well-established protein that is processable into fibers, [6,7] films, [8,9,10,11] foams, particles, [12] hydrogels, [13,14], and, recently, aerogels after supercritical drying with CO_2_ [15,16,17]. The production of silk fibroin-based materials requires a detailed understanding of the solvent-mediated dielectric environment to induce the hierarchical self-assembly responsible for the mechanical properties resulting from protein primary structure and molecular assembly [18]. Particularly, in silk fibroin, there are three major folding secondary structure motifs, including random coils, alpha helices, or beta-sheets, which control the silk’s strength [19]. Specifically, silk fibroin molecular folding can be controlled by the solvent, which forces its primary structure to arrange into the above mentioned secondary structures [18,20].

Alcohols as solvents are useful to stabilize specific secondary structures of silk fibroin in aqueous environments while denaturing the native tertiary conformation [21]. Because the properties of silk fibroin depend strongly on the preparation conditions (fluid environment), the choice of solvent affects the overall quality of the bulk material and the formed nanostructure [3]. 1,1,1,3,3,3-Hexafluoro-propan-2-ol (HFIP) is one of the most applicable solvents for the stabilization of silk’s secondary structures, particularly the alpha-helical conformation [21,22,23,24,25,26]. The high polarity of HFIP as an alcohol allows it to stabilize the silk helical state by decreasing the polarity of the protein chains. This results in local hydrogen bonds that stabilize amphiphilic helical conformations, producing a silk alcogel [21]. The protein concentration is a significant factor in unfolded conformations as the silk secondary structure switches between random coil and alpha-helix depending on the different molecular interactions that can occur [21].

Silk fibroin exposed to HFIP or other alcohols has been used to make gel materials, which can be used for fracture fixation systems or artificial fibers after convective drying [18,26,27]. Dissolving silk in HFIP to cast different forms, particularly gel materials, before convective drying confers the mechanical properties of the folded protein structure and maintains the assembled bulk solid structure in the alcogel formation [27]. Additionally, controlling the nanostructure of the silk fibroin, nanofibrils has been achieved with supercritical CO_2_ drying (SCCO_2_) [17]. Supercritical drying ensures the porosity of the material and maintains the high surface area and low density [28,29,30]. Using this drying method results in molecular conformational changes of the HFIP–silk fiber, which produce stronger, high surface area materials that have potential for novel biomedical applications. 

Furthermore, enhancing the properties of biopolymer gels, such as metallization for catalysis and sensing, can be achieved by equilibrating with gold, palladium, and platinum noble metal complexes, which, after electrochemical reduction, result in nanoparticle growth on the biopolymer nanofibrils [4,5]. Using silk fibroin as the material of choice to add conductive noble metal nanoparticles would enhance the versatility of this mechanically robust and biocompatible material. 

Here, we demonstrate the preparation of a composite material consisting of noble metal nanoparticles attached to HFIP-treated porous silk aerogel fibers forming a composite material. Maintaining a constant concentration but changing the type of noble metal ion species determines the extent of the nanoparticle growth on the silk nanofibril surface along with the resulting percentage of metal content in the final aerogel fiber. This allows for material variation based on nobility compared with concentration. The controllable bulk geometries improve this platform to allow it to be used for biofibers. Lastly, utilizing different noble metals can extend the applicability of these biopolymer nanofibrils for other applications, such as catalysis, energy storage, and sensing.

## 2. Materials and Methods

### 2.1. Silk Fibroin Fiber Aerogel Synthesis

Silk fibroin solution was prepared as previously described [31]. *B. mori* silkworm cocoons were boiled for 30 minutes in a solution of 0.02 M Na_2_CO_3_ to remove the sericin glycoprotein. The extracted fibroin was rinsed in deionized water and dried at ambient conditions for 12 h. The dried fibroin was dissolved in 9.3 M LiBr solution at 60 °C for 3 h. The solution was dialyzed against deionized water using a dialysis cassette (Slide-a-Lyzer, Pierce, molecular weight cut-off (MWCO) 3.5 kDa) at room temperature for 2 days until the solution reached a concentration of approximately 60 mg/mL. The obtained solution was purified by centrifugation (20 min at 11,000× *g*) to remove large aggregates.

For the HFIP aerogels, reconstituted silk fibroin was frozen and lyophilized, resulting in a dried material. The dried silk was stored in ambient conditions to prevent any rehydration of the lyophilized silk fibroin. The lyophilized silk was resolubilized in 1,1,1,3,3,3-Hexafluoro-propan-2-ol (HFIP) (Matrix Scientific, Columbia, SC, USA) to generate a 40 mg/mL solution. The concentration is critical as solutions with lower concentrations produce aggregated gels. The solubilized HFIP silk solution was stored at ambient temperatures until used in the gel-forming process. 

The silk aerogels were prepared using silicon tubing with an inner diameter of 1.5 mm (McMaster-Carr, Robbinsville, NJ, USA) and filled with HFIP/silk solution. After the HFIP/silk solution was put into the silicon tubing, the HFIP-silk was submerged in 200 proof ethanol (Fisher Scientific, Waltham, MA, USA). Ethanol diffusion into the HFIP-silk proceeded for 24 h to induce physical crosslinking and form a free-standing fiber gel. Additional ethanol rinses were performed to displace the HFIP after the silk gel fiber was removed from the silicon tubing.

After physical crosslinking, the silk fiber gels were rinsed in deionized water and equilibrated in 100 mM of sodium tetrachloropalladate (II) (Na_2_PdCl_4_), potassium tetrachloroplatinate (II) (K_2_PtCl_4_), or gold chloride trihydrate (HAuCl_4_·3H_2_O) (Sigma Aldrich, St. Louis, MO, USA) for 24 h.

The fiber samples were reduced in 100 mM sodium borohydride for 24 h for noble metal nanoparticle growth [32]. The silk–metal composite gels were rinsed in deionized water for 24 h to remove excess reducing agent. To maintain the metal-coated nanofibrillar hydrogel network, samples were then dehydrated in a series of ethanol rinses at concentrations of 25, 50, 75, and 100% for 30 min each and then supercritically dried in CO_2_ using a Leica EM CPD300 Automated Critical Point Dryer (Buffalo Grove, IL, USA) with a set point of 35 °C and 1200 psi.

To prepare sufficient sample material for X-ray diffraction, thermal gravimetric, and nitrogen gas adsorption analysis, silk–metal composites were prepared in a bulk monolith geometry. A HFIP–silk solution was cast in a 48-well cell culture dish (diameter of 10 mm) and crosslinked with 200 proof ethanol by casting on the top for 24 h. After crosslinking, silk hydrogels were rinsed in deionized water for 48 h to remove any remaining HFIP and ethanol. The hydrogels were equilibrated in 100 mM of sodium tetrachloropalladate (II) (Na_2_PdCl_4_), potassium tetrachloroplatinate (II) (K_2_PtCl_4_), or gold chloride trihydrate (HAuCl_4_·3H_2_O) as in the case of the silk–metal fiber synthesis above but for 48 h. To ensure reduction throughout the volume of the silk gels, 2 M sodium borohydride (NaBH_4_) and 2 M dimethylamine borane (DMAB) was used for the palladium-, and platinum- and gold-equilibrated silk gels, respectively. The high reducing agent concentration was used to drive diffusion into the depth of the gel. Electrochemical reduction proceeded for 24 h before rinsing for 48 h in deionized water. An ethanol solvent exchange was performed prior to supercritical drying in CO_2_.

### 2.2. Scanning Electron Microscopy (SEM)

SEM was used to evaluate scaffold morphology. All the micrographs were taken with a TM-3000 Scanning Electron Microscope (Hitachi, Tokyo, Japan) or a FEI Helios 600 scanning electron microscope (ThermoFisher Scientific, Hillsboro, OR, USA). Samples were not coated in gold prior to imaging.

### 2.3. X-ray Diffractometry (XRD)

XRD measurements were performed using a PANalytical Empyrean (Malvern PANalytical, Almelo, The Netherlands) diffractometer with scans at 45 kV and 40 mA with Cu K_α_ radiation (1.54060 Å), a 2θ step size of 0.0130°, and 20 s per step for diffraction angles (2θ) performed from 5° to 90°. XRD spectra analysis was performed with High Score Plus software (Malvern PANalytical, Almelo, The Netherlands). Crystallite size (D) was determined with the Debeye–Scherrer formula D = Kλ(Bcosθ)^−1^ with the shape factor (K), full width at half maxima (B), radiation wave length (λ), and Bragg angle (θ). A shape factor of K = 0.9 was used. High Score Plus software was used to analyze the XRD spectra (Version 4.6, Malvern PANalytical, Almelo, The Netherlands).

### 2.4. Thermal Gravimetric Analysis (TGA)

Thermal gravimetric analysis (TGA) was performed on a Thermal Instruments Q-500 (New Castle, DE, USA) in a ramp state with a temperature rate of 10 °C/min from ambient to 1000 °C. Samples were maintained under nitrogen gas flow at a rate of 60 ml/min.

### 2.5. Fourier Transform Infrared (FTIR) Spectroscopy

FTIR analysis of silk film samples was performed in a PerkinElmer Frontier Optica FIR spectrometer (PerkinElmer, Waltham, MA, USA) in attenuated total reflectance (ATR). Films were measured before and after the galvanic displacement. For each sample, 64 scans were collected with a resolution of 1 cm^−1^, with a wave number range of 4000–650 cm^−1^.

### 2.6. Porosity and Surface Area Analysis

Nitrogen gas adsorption–desorption measurements were performed according to International Union of Pure and Applied Chemistry (IUPAC) standards [33] using a Micromeritics ASAP 2020 Plus (Micromeritics, Norcross, GA, USA) to determine surface area and pore size. All the samples were vacuum degassed at 100 °C for 10 h prior to analysis. Brunauer–Emmett–Teller (BET) analysis [34] was used to determine the specific surface area from gas adsorption. Pore size distributions for each sample were calculated using the Barrett–Joyner–Halenda (BJH) model [35] applied to volumetric desorption isotherms. All the calculations were performed using Micromeritics’ ASAP 2020 software (Micromeritics, Norcross, GA, USA). 

## 3. Results and Discussion

### 3.1. Silk Fibroin Aerogel Synthesis

**Figure 1:**
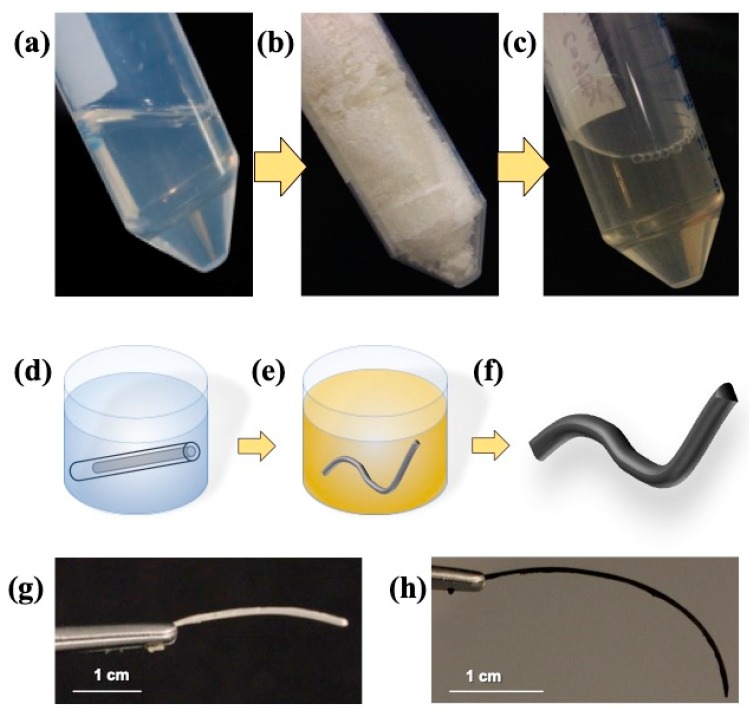
Silk fibroin aerogel fiber synthesis scheme. Different working solutions of silk fibroin starting with (**a**) regenerated silk fibroin solution, (**b**) lyophilized silk fibroin, and (**c**) hexafluoro-2-propanol (HFIP)–silk fibroin. Scheme depiction of the synthesis of noble metal silk fibroin aerogel fibers. (**d**) HFIP–silk fibroin in silicon tubing mold in an ethanol bath to induce physical crosslinking, (**e**) equilibrating in noble metal ionic solution, and (**f**) after reduction and supercritical drying. (**g**) The silk fibroin aerogel fiber without noble metal addition and (**h**) the silk–palladium aerogel fiber (scale bars are 1 cm).

A fast and robust method was developed to form noble metal composite silk fibroin nanostructured fibers by crosslinking HFIP–silk solution with ethanol. The average molecular weight of the silk was 100 kDa and a concentration of 40 mg/mL. Figure 1 shows the synthesis scheme for the silk fibroin noble metal composite aerogels. In order to physically crosslink the silk, the HFIP–silk solution was injected into a silicon tubing mold with an inner diameter of 1.5 mm and 3 cm long and submerged in a bath of 100% ethanol (Figure 1c–d). Parameters such as silk fibroin concentration, noble metal concentration, and reducing agent concentration were the determining factors in the composite fibers. The type of noble metal dictated the noble metal particle size and allowed for particles less than 10 nm and noble metal deposition onto the silk template. Visible deposition of noble metal nanoparticles was observed by a visible color change compared with a control (Figure 1g–h). The formed fibers after reduction provided flexibility and were able to bend into geometric shapes. 

To demonstrate the ability to deposit multiple noble metals, silk hydrogels were prepared as cylindrical gels and equilibrated in 100 mM noble metal ion solutions of palladium (Na_2_PdCl_4_) and platinum (K_2_PtCl_6_) (Figure S1). Reduction of the metal ions can occur from multiple reducing agents, such as DMAB or NaBH_4_, as represented by palladium reduction and platinum reduction in Figure S1c,d, respectively. Furthermore, 100 mM metal ion solutions of gold (HAuCl_4_) were also reduced in DMAB (Figure S2a–c). The noble metal type and reducing agent changes the morphology of the aerogel fibers as visible in SEM images (Figures S3 and S4). The silk–gold aerogels reduced in DMAB show thinner nanofibrils (30–50 nm) with smaller pore sizes compared with the silk–platinum aerogels reduced in NaBH_4_, which have larger diameter nanofibrils (70–100 nm) and larger pores. 

### 3.2. Aerogel Morphology and Noble Metal Nanoparticles

**Figure 2:**
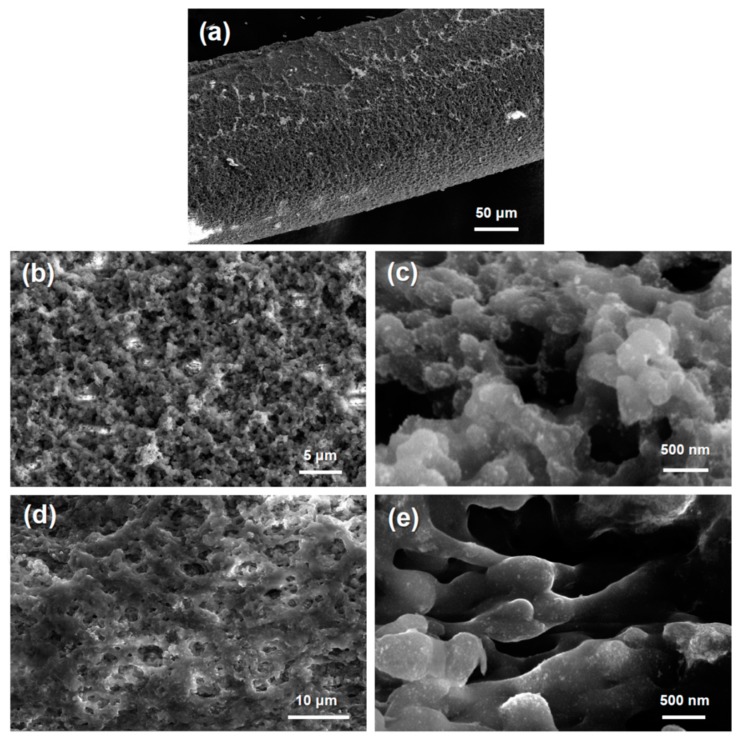
Scanning electron microscopy images. (**a**–**c**) The silk–palladium composite aerogel fibers. (**d**–**e**) The silk–platinum composite aerogel fibers.

The structure of the nanofibrils and growth of the noble metal nanoparticles indicates the effects of reduction on the silk fibroin. It has been previously demonstrated that the reduction of these noble metals can be completed using gelatin and cellulose materials with higher reducing agent concentrations [4,5]. Figure 2 shows the SEM images of the silk fibroin aerogel with platinum and palladium after reduction with 100 mM sodium borohydride. Figure 2a shows the fiber formation of the HFIP–silk aerogel fiber. At higher magnification, the presence of nanoparticles covers the silk fiber network for the silk–palladium composite aerogel fibers (Figure 2b–c). This is observed by the brighter regions indicative of nanoparticle growth. The silk–platinum composite aerogel fibers show a more even distribution of noble metal nanoparticle growth onto the silk fibroin (Figure 2d–e).

The morphology of the underlying HFIP–silk fibroin template shows an interconnected network of silk protein spherical particles with diameters of ~500 nm forming a string of pearl-like fibers with high porosity, which is caused by the gelation of the original HFIP–silk (Figure S3). The interconnected network is visible at higher magnification in the anchored silk–gold composite aerogel fibers (Figure S4a–c) when reduced with DMAB. The gold nanoparticles cluster around the larger silk nanofibrils in discrete nanoparticles. Chemical reduction with sodium borohydride of the silk–platinum composite aerogels shows an interconnected silk nanofibril network with nanoparticles dispersed on the surface with a diameter in the range of 5–20 nm (Figure S4d–f).

The change in fiber morphology is possibly caused by the changes in pH of the noble metal ion solution where solutions of HAuCl_4_ have lower pH values compared with K_2_PtCl_6_ or Na_2_PdCl_4_. Due to the lower pH, gold metal ions are more attracted to the silk molecular structure caused by electrostatic interactions, which is visible as a higher density of decorated surface nanoparticles compared with the silk–platinum and silk–palladium aerogels (Figure S3). This has been discussed in other biopolymer-related work [4,5]. Silk fibroin has a repeating pattern of glycine, serine, and alanine forming polymer blocks with shorter block regions of non-repeating sequences [36]. It is these amino acids that allow silk to grow noble metal nanoparticles.

### 3.3. XRD Characterization

**Figure 3:**
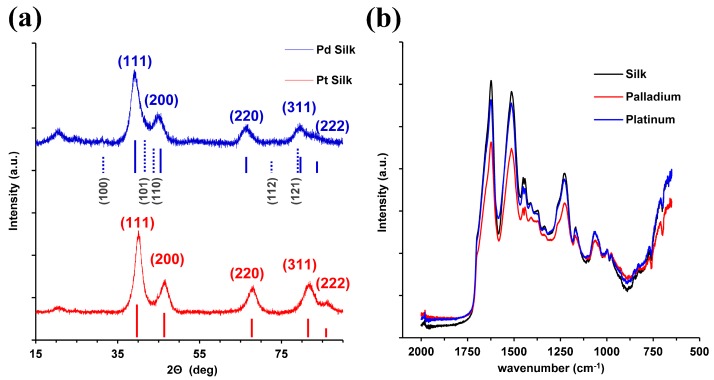
(**a**) X-ray diffraction spectra for silk palladium and platinum composite aerogels. The silk–palladium aerogel peaks are indexed to the Joint Committee on Powder Diffraction Standards (JCPDS) reference 01-087-0637 for palladium (blue lines), 01-073-0004 for palladium hydride (blue dashed lines; Miller indices labeled in gray). The silk–platinum aerogels are indexed to 00-004-0802 (red lines) for platinum. (**b**) Fourier transform infrared (FTIR) spectra for the silk, silk–palladium, and silk–platinum fiber aerogels.

Figure 3a shows the X-ray diffraction (XRD) spectra for the HFIP–silk composite aerogel composites synthesized with palladium and platinum, respectively. XRD spectra for aerogel composites prepared with palladium were indexed to the Joint Committee on Powder Diffraction Standards (JCPDS) reference number 01-087-0637 for palladium and 01-073-0004 for palladium hydride. For aerogel composites prepared with platinum, XRD peaks were indexed to JCPDS reference number 00-004-0802 for platinum. Both palladium and platinum phases are cubic crystal systems with Fm-3 m space groups. The shape evolution of the palladium and platinum nanocrystals of different morphologies can be directed by agents, such as silk and other secondary chemicals, during reduction [37]. The minor presence of palladium hydride in the Pd–silk aerogel composites is likely due to hydrogen gas evolution during electrochemical reduction and the tendency of palladium to store hydrogen gas within its crystal lattice [38]. The palladium hydride peaks shift the position of the fitted palladium phase peaks slightly from their indexed positions. For instance, the (111) palladium fitted peak at 39.5° is shifted right relative to the indexed reference peak position at 39.0° likely due to peak convolution with the (101) palladium hydride peak. The change in nanocrystal structure can be associated with the shape-directing capabilities of the hydrogen evolution during reduction, which is observed as the visible peak shift in the XRD spectra [37]. The crystallite sizes, determined by using the Debeye–Scherrer formula, and the (111) peaks were 3.6 nm and 2.6 nm for silk–palladium and silk–platinum, respectively, and suggest that the nanoparticles observed in the SEM images in Figure 2 are polycrystalline. The XRD spectra for the HFIP–silk composite aerogel fiber with gold is shown in Figure S5a. The peaks observed for both the silk–palladium and silk–platinum composite aerogels at approximately 20.8° are attributed to the silk protein templates [39,40,41].

### 3.4. FTIR Characterization

The secondary structure of the silk fibroin was completed by FTIR, examining the secondary structure in the Amide II and III band. The FTIR spectra for silk, silk–palladium, and silk–platinum are shown in Figure 3b [42]. The Amide I band is associated with 1600–1700 cm^−1^, which shows the characteristic peak of beta-sheeted silk fibroin at 1625 cm^−1^ [20]. The alpha-helix structure (1658–1662 cm^−1^) additionally shows a sharp peak associated with the shoulder near the Amide I band [20]. The FTIR spectra are characteristic of silk and are unchanged for the silk–palladium and silk–platinum aerogel fibers. After supercritical drying there is a high percentage of alpha-helix and beta-sheet content through the interaction at the molecular level. The same was found in the silk–gold aerogel fibers (Figure S5b). The high beta-sheet content observed is typical of HFIP–silk and has been shown in other studies where silk gels dried in ethanol or methanol are used for fracture fixation devices [27].

### 3.5. TGA Characterization

**Figure 4:**
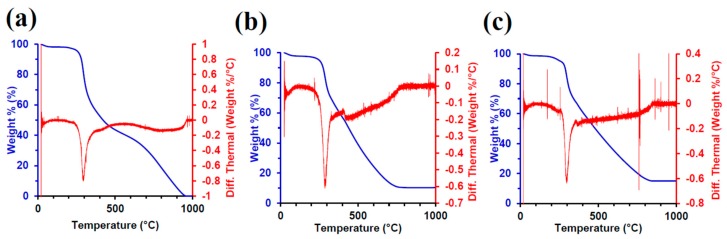
Thermogravimetric analysis (TGA) with differential thermal analysis (DTA) for the (**a**) silk aerogel, (**b**) silk–palladium aerogel, and (**c**) silk–platinum aerogel.

To characterize the mass composition of noble metals in the silk aerogels, thermogravimetric analysis (TGA) was performed with the results shown in Figure 4. When examined, the control silk, silk–palladium, and silk–platinum aerogel fibers showed an increase in the metal-to-silk weight ratio. Silk begins decomposition above 200 °C, but to ensure the complete degradation of the silk fibroin from the sample, analysis was conducted up to 1000 °C. The final mass percentage indicates the amount of noble metal to silk fibroin. The silk-only control sample shows 0% metal at 1000 °C [20,43,44], silk–palladium shows 10%, and silk–platinum indicates a mass percentage of 15%. The silk–gold aerogel fiber composites showed a higher metal mass percentage of 30%, which was observed in the morphological changes in the SEM images described above (Figure S6). The change in mass percentage of noble metal varied between each condition as stated above due to the electrostatic forces between the metal ions and silk fibroin. As stated above, the different pH values for 100 mM Na_2_PdCl_4_, K_2_PtCl_4_, or HAuCl_4_·3H_2_O cause different ionic interactions with the silk. Lower pH will deposit more metal ions, which, after reduction, shows a higher density of nanoparticles on the silk surface and is consistent with the mass percent differences observed with TGA.

### 3.6. Porosity and Surface Area Characterization

**Figure 5:**
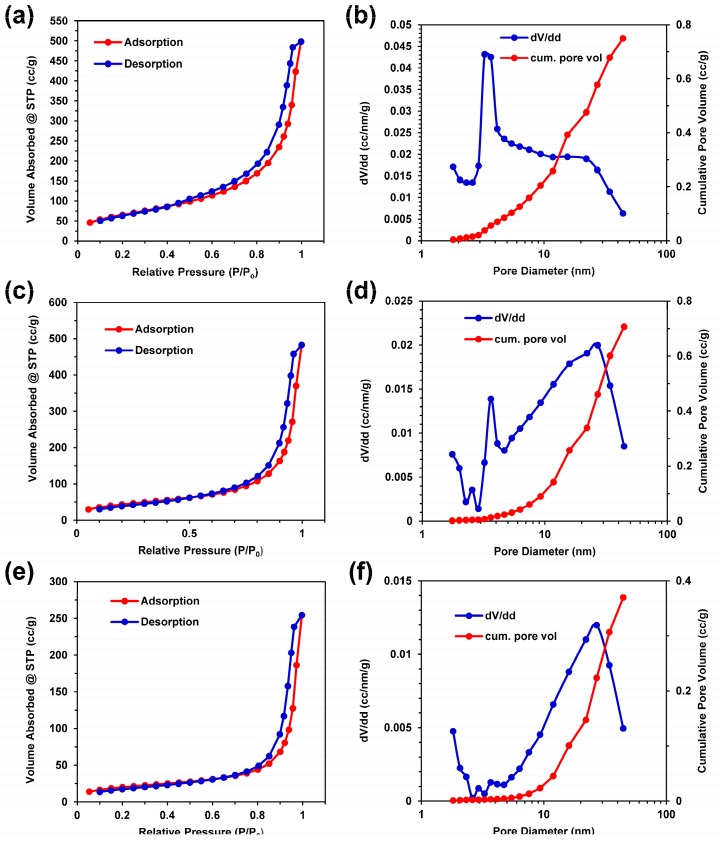
Nitrogen adsorption–desorption isotherms and pore size distribution with cumulative pore volume for the (**a**,**b**) silk aerogels, (**c**,**d**) palladium–silk aerogels, and (**e**,**f**) platinum–silk aerogels.

Nitrogen gas adsorption isotherms were generated for the silk–noble metal aerogels prepared with 100 mM noble metals. Given the small mass of the silk aerogel composite fibers, bulk silk and silk composite aerogels were used to achieve nitrogen gas adsorption–desorption isotherms. The physisorption data shown in Figure 5 indicate type IV adsorption–desorption isotherms according to IUPAC classification standards, suggesting the presence of both mesoporous (2–50 nm) and macroporous (>50 nm) structures in all of the aerogel samples. The macropore features generally correlate with the pores observed in the SEM images in Figure 2 for the silk–palladium and silk–platinum composites. The mesoporous content suggests porosity within the silk phase of the composite aerogels not directly observed with SEM. After a sharp rise in adsorbed gas, no limiting adsorption plateau at high relative pressures (P/P_o_) is observed. The maximum volume adsorbed at the highest relative pressure of P/P_o_ = 0.995 is 498, 482, and 254 cm^3^/g for the silk, silk–palladium, and silk–platinum samples, respectively. All the sample isotherms exhibit type H3 hysteresis typical of mesoporous capillary condensation. The hysteresis loops close at higher relative pressures for the silk–palladium and silk–platinum composite aerogels compared with the silk only aerogels. This may be due to metal nanoparticles filling or blocking the smaller pores. This is supported by the pore frequency distributions seen in Figure 5b,d,e for silk, silk–palladium, and silk–platinum, respectively. The silk aerogels exhibit a high frequency of 3–4 nm pores, with a broad presence of mesopores up to 40 nm. The frequency of 3–4 nm pores decreases for the silk–palladium and silk–platinum aerogels with a commensurate increase in mesopores of diameters of 20–30 nm. The BET specific surface areas determined from desorption isotherms of the silk control, silk–palladium, and silk–platinum samples were 268, 170, and 72 m^2^/g, respectively. The decrease in specific surface area from pure silk to silk–metal composite aerogels is consistent with nanoparticle pore blockage, which is consistent with SEM image analysis.

## 4. Conclusions

Here we have shown that HFIP–silk fibroin aerogel fibers can be utilized as a platform to anchor noble metal nanoparticles onto the surface of silk nanofibrils. The silk aerogels have a high surface area and are composed of different percentages of noble metals, which are based primarily on the nobility, not the concentration. This changes the mass percentage, porosity, surface area, and pore diameter. Compared with other biopolymers, which have acted as templates with noble metals, silk is much simpler to process and does not require any chemical crosslinking. Palladium, platinum, and gold nanoparticle growth have been shown on silk fibroin aerogel fibers. The molecular structure of silk is unchanged when anchored with different noble metals. These silk fibroin noble metal aerogels have potential utility in energy storage and conversion that can be used where degradable, flexible materials are required.

## 5. Patents

This work has been submitted with a preliminary patent and invention disclosure.

## Supplementary Materials

The following are available online at https://www.mdpi.com/1996-1944/12/6/894/s1, Figure S1: Silk hydrogels equilibrated in 100 mM Na_2_PdCl_4_ and 100 mM K_2_PtCl_6_ reduced in 2 M NaBH_4_; Figure S2: Silk hydrogels equilibrated in 100 mM HAuCl_4_ reduced with 2 M DMAB; Figure S3: SEM images of the HFIP–silk noble metal fibers; Figure S4: SEM images of the HFIP–silk aerogels for silk–palladium, silk–platinum, and silk–gold; Figure S5: X-ray diffraction (XRD) and Fourier transform infrared (FTIR) absorption spectra for the silk–gold composite aerogels, Figure S6: TGA curves of the silk–gold aerogels; Figure S7: Nitrogen gas adsorption–desorption curves for the silk–gold aerogels.
