# Supplementary material for: Noble Metal Composite Porous Silk Fibroin Aerogel Fibers
Source: Materials (Basel). 2019 Mar 18;12(6):894. doi: 10.3390/ma12060894 (PMC6470705; doi:10.3390/ma12060894)
Supplement: Supplementary file 1 [file materials-12-00894-s001.pdf]

materials

Noble metal composite porous silk fibroin aerogel fibers

Alexander N. Mitropoulos [1,2,*], F. John Burpo [1,*], Chi K. Nguyen [1], Enoch A. Nagelli [1], Madeline Y. Ryu [1], Jenny Wang [1], R. Kenneth Sims [3], Kamil Woronowicz [1] and J. Kenneth Wickiser [1]

1 Department of Chemistry and Life Science, United States Military Academy, West Point, NY 10996 USA; chi.nguyen@westpoint.edu (C.N.), enoch.nagelli@westpoint.edu (E.A.N.), madeline.ryu@westpoint.edu (M.R), jenny.wang@westpoint.edu (J.W.), kamil.woronowicz@westpoint.edu (K.W.), ken.wickiser@westpoint.edu (J.K.W.)
2 Department of Mathematical Sciences, United States Military Academy, West Point, NY 10996 USA; alexander.mitropoulos@gmail.com
3 Department of Civil and Mechanical Engineering, United States Military Academy, West Point, NY 10996 USA; robert.sims@westpoint.edu (R.K.S.)
* Correspondence: alexander.mitropoulos@gmail.com (A.N.M.); john.burpo@westpoint.edu (F.J.B.); Tel.: +01-845-938-3900 (F.J.B.)

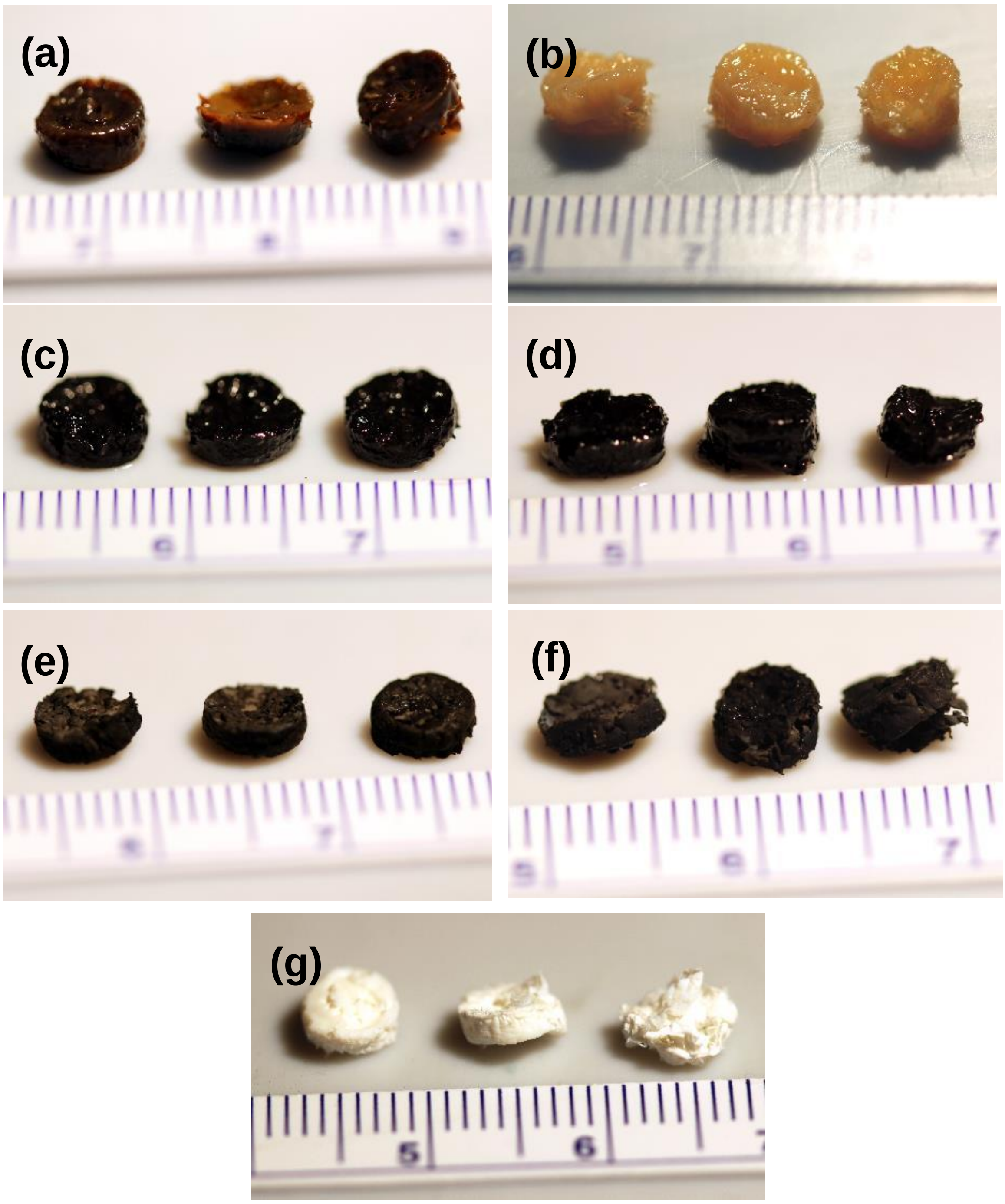

Figure 1. Aerogel synthesis process photos. Silk hydrogels equilibrated in **(a)** 100 mM Na_2PdCl_4 and **(b)** 100 mM K_2PtCl_6 (in 1:1 deionized water to ethanol solvent). **(c)** Hydrogel from (a) reduced with 2 M DMAB. **(d)** Hydrogel in (b) reduced with 2 M $NaBH_4$. Supercritically dried aerogels: **(e)** silk-palladium, **(f)** silk-platinum, and **(e)** silk only.

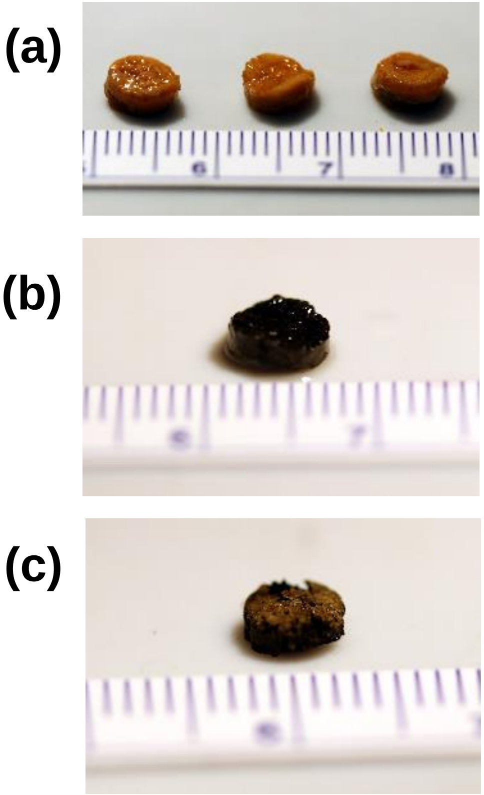

Figure 2. Aerogel synthesis process photos. Silk hydrogels equilibrated in **(a)** 100 mM $HAuCl_4$, **(b)** reduced with 2 M DMAB. **(c)** Supercritically dried silk-Au aerogel composite.

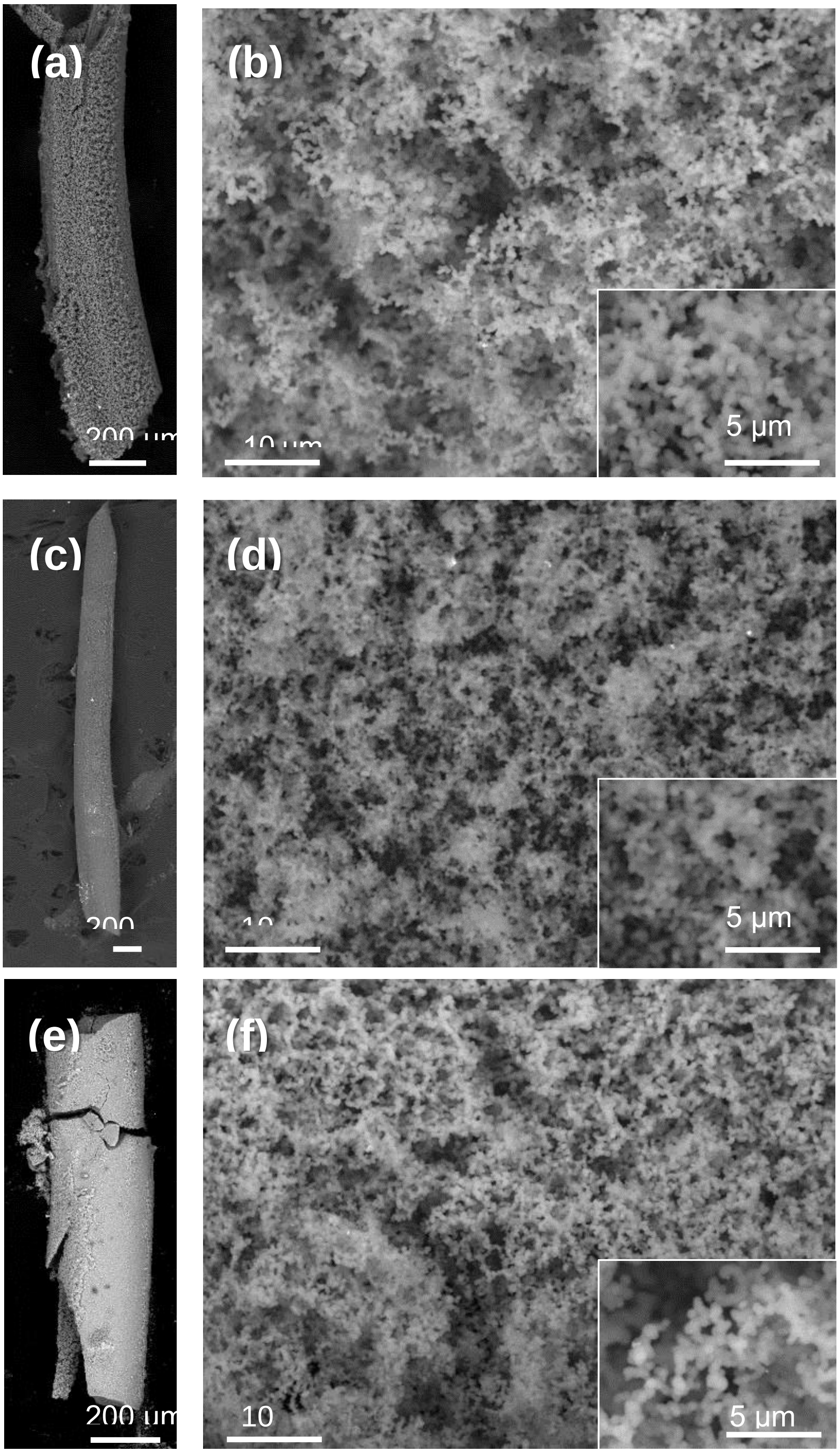

Figure 3. HFIP-silk noble metal fibers. **(a)-(b)** Silk-platinum composite aerogel fibers, **(c)-(d) s**ilk-palladium composite aerogel fibers, and **(e)-(f)** silk-gold composite aerogel fibers.

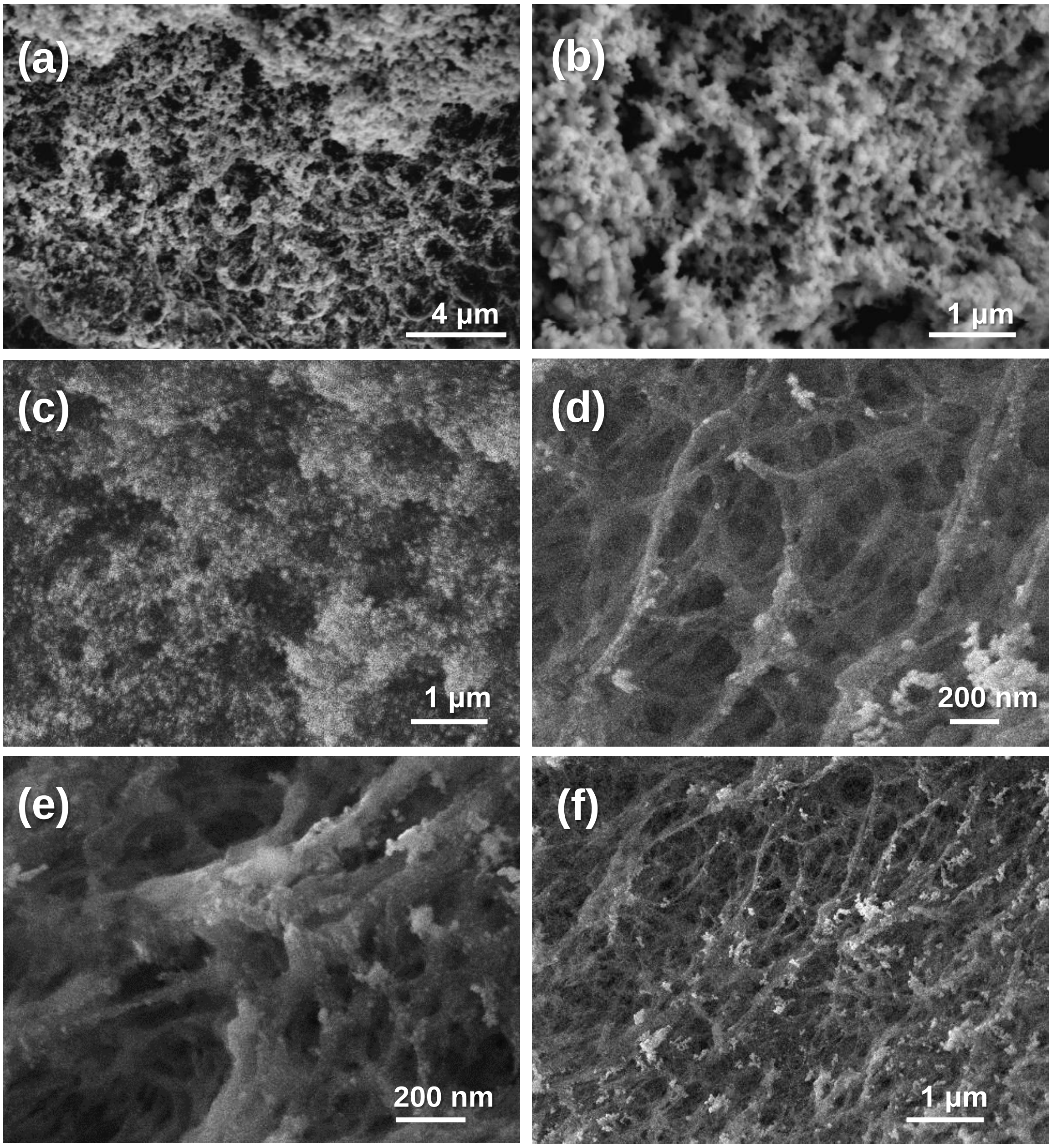

Figure 4. Scanning electron microscope images of **(a)-(c)** silk-gold composite fibers, and **(d)-(f)** silk-platinum composite aerogels.

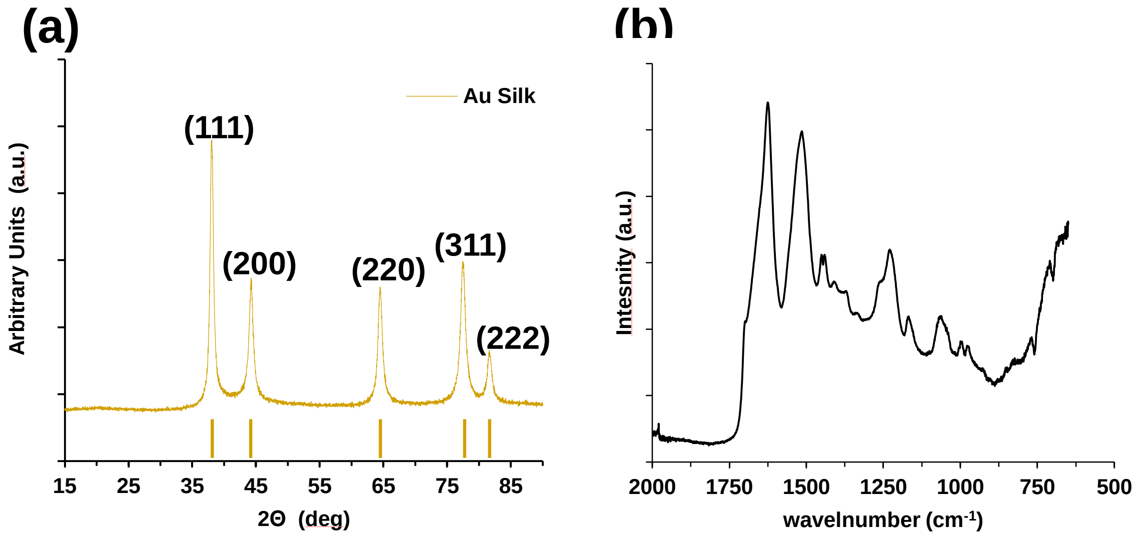

Figure 5. **(a)** X-ray diffraction (XRD) spectra for silk-gold aerogels with peaks indexed to JCPDS reference 01-071-4614 for gold (gold lines). **(b)** Fourier transform infrared (FTIR) absorption spectra for silk-gold fiber aerogels.

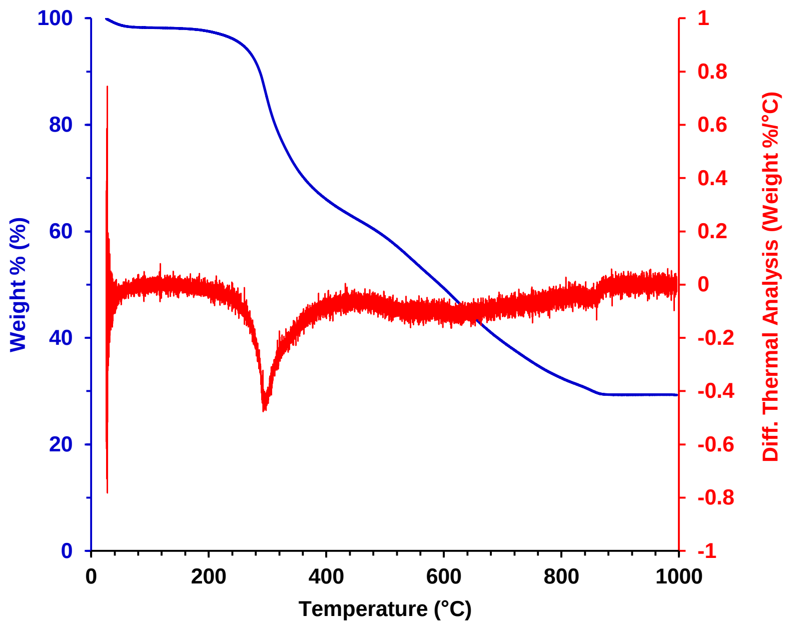

Figure 6. Thermal gravimetric analysis (TGA) – silk gold aerogel composite.

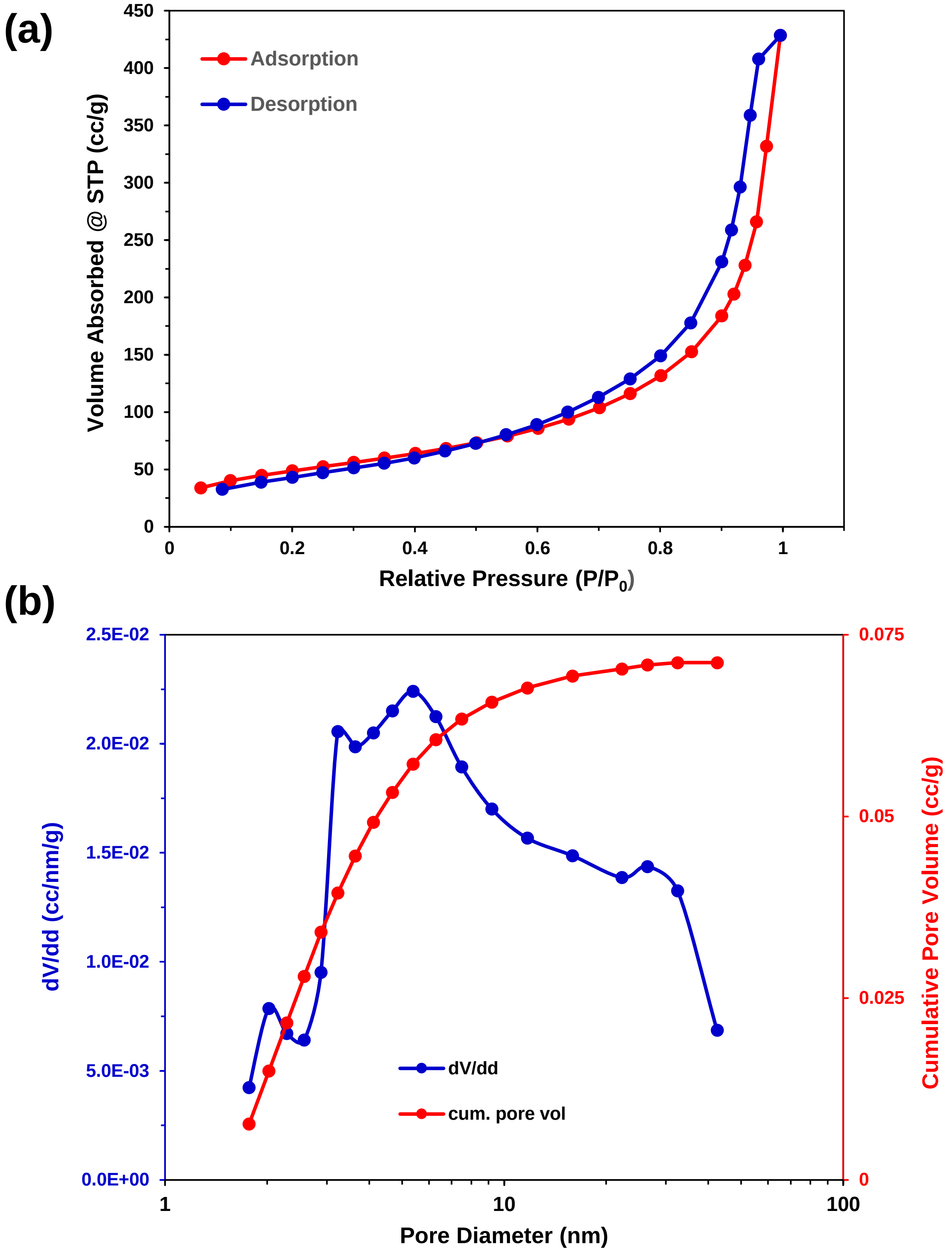

Figure 7. Silk-Au composite aerogel **(a)** nitrogen adsorption-desorption isotherm, and **(b)** pore size distribution with cumulative pore volume.
